# Reconstruction of Soft Tissue Defect With a Free Vascularized Anterolateral Thigh Flap After Resection of Soft Tissue Sarcoma in Extremities

**DOI:** 10.1111/os.12840

**Published:** 2021-12-13

**Authors:** Jun Qiao, Haijun Mao, Li Wen, Leilei Xu, Zezhang Zhu, Yong Qiu, Jin Xiong, Shoufeng Wang

**Affiliations:** ^1^ Department of Orthopedic Surgery The Affiliated Drum Tower Hospital of Nanjing University Medical School Nanjing China

**Keywords:** Defect, Free vascularized anterolateral thigh flap, Reconstruction, Soft tissue sarcomas

## Abstract

**Objective:**

This study aims to determine outcomes and complications in functional reconstruction of soft tissue defects after surgical resection for soft tissue sarcomas (STSs) of extremities.

**Methods:**

A retrospective chart review was performed on patients with STSs of extremities from May 2015 to April 2019 who underwent radical resection of STSs and reconstruction of soft tissue defect with free vascularized anterolateral thigh flap (FVALTP). A minimum 3‐month follow‐up was required for all the patients. Patient demographics and comorbidities, flap characteristics, postoperative complications, and time to heal were recorded. The functional outcomes of the reconstructed limbs were assessed by the Musculoskeletal Tumour Society（MSTS） scoring system.

**Results:**

A total of 11 patients (four males and seven females) were included in the study. The mean age was 62 years (range: 29–84 years). The mean surface area was 151.4 cm^2^ (range: from 64 cm^2^ to 418cm^2^). The mean operation time was 126 min (range: 95–296 min). The mean follow‐up was 17.5 months (range: 6–34 months). The mean score of MSTS at last follow‐up was 26.2 (range: 12–29). Incision healed by first intention in eight patients. Incision healed by second intention in three patients. A patient who had received preoperative radiotherapy experienced delayed union. After debridement, the patient successfully got union. Another two patients experienced marginal necrosis of flap due to vascular crisis. After 3‐week dressing changes, the patients also got satisfactory union. One case suffered from vascular crisis during surgery in which the procedure was changed into skin grafting to cover resection site.

**Conclusion:**

FVALTP technique can be effectively applied to the reconstruction of soft tissue defect after STSs resection. The short‐term follow‐up indicated satisfactory functional outcome and low incidence of previously known complications. It was necessary to further validate its efficacy in reconstruction of soft tissue defect after malignant extremity soft tissue sarcoma resection.

## Graphical abstract

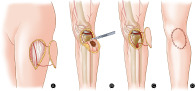

Soft tissue sarcomas (STSs) represent 1% of adult and 7%–15% of pediatric malignancies^1^. Its incidence is estimated at 4–5/100.000/year^2^. As a heterogeneous group of tumors of mesenchymal origin, they can occur anywhere in the body, with the extremities being the most common primary site^3^, ^4^. Amputation was once the mainstay for the treatment of STS with the advantage of negative margins and low incidence of reoccurrence. However, it compromises the patient's everyday routine by limiting self‐sufficiency. Limb shortening is also responsible for an asymmetric gait and posture deformities. With the development of surgical techniques and radiotherapy, limb‐sparing surgery is considered as first choice in most patients with soft tissue sarcomas of the extremities. The standard curative treatment for extremity STS is surgical resection with negative margins and radiotherapy for intermediate and high‐grade sarcomas. Müller^5^ conducted a large retrospective study including 769 patients with a high‐grade soft tissue sarcoma of the extremities, who underwent a limb‐sparing surgery, and found a local recurrence‐free survival of 83.2% after 5 years and 75.9% after 10 years. Negative surgical margin is of great importance for controlling reoccurrence. Novais^6^ retrospectively reviewed the medical records of 248 patients who had soft tissue sarcomas of the extremities treated surgically from 1995 to 2008 and found patients who presented with positive margins or a margin of 2 mm or less had a worse survival than patients who had margins of greater than 2 mm and wide margins (5‐year survival, 47% *vs* 70% and 72%). Adequate resection range is the premise of getting negative margins^7^. One of the challenges for surgeons was soft tissue defect after surgical resection of sarcoma, and various reconstruction techniques have been employed to address this issue, including the latissimus dorsi free flap, dorsalis pedis free flap, scapular free flap, lateral arm free flap, and anterolateral thigh (ALT) free flap. Free vascularized anterolateral thigh flap (FVALTP) has been widely used for reconstruction of soft tissue defects all over the body^8^. The anterolateral thigh flap can be harvested with a multitude of tissue components including skin, fat, muscle, and fascia. The anterolateral thigh flap can be designed with a significant fascial component in the tensor fasciae latae, which is advantageous considering the recipient site in question^9^. The aim of this study was to investigate outcomes and complications in functional reconstruction of soft tissue defects after surgical resection for STSs of extremities.

## Materials and Methods

A retrospective chart review was performed on patients with STSs of extremities from May 2015 to April 2019. The inclusion criteria were as follows: (i) receiving radical resection and reconstruction with FVALTP (Table 1); (ii) histologically diagnosed as STS; and (iii) with a minimum of 3‐month follow‐up. Patient demographics and comorbidities, flap characteristics, postoperative complications, and time to heal were recorded.

**TABLE 1:** Demographic data of the patients

No.	Gender	Age	Diagnosis	Location	Complication	Management of complications	Follow‐up (Months)
1	F	54	UPS	Posterior aspect of lower limb	Without		34
2	M	71	Fibrosarcoma	Anteromedial knee	Without		6
3	F	70	Recurrent UPS	Anterolateral knee	Without		16
4	M	61	Recurrent liposarcoma	Anterolateral thigh	Without		23
5	F	67	UPS	Posterior knee	Without		21
6	M	29	Malignant peripheral nerve sheath sarcoma	Lateral side of left foot	Delayed healing	debridement and dressing change	16
7	F	84	UPS	Posterior knee	Vascular crisis	changed into skin grafting	9
8	F	65	UPS	Anteromedial knee	Delayed healing	dressing change	25
9	F	43	Malignant melanoma	Lower limb	Delayed healing	dressing change	13
10	F	65	Liposarcoma	Posterior thigh	Without		17
11	M	71	Synovial sarcoma	Anteromedial knee	Without		12

Abbreviation: UPS, undifferentiated pleomorphic sarcoma.

### Operative Technique

#### Anesthesia and Position

The procedure was performed with the patient in supine position and under general anesthesia.

### Surgical Procedure

**FIG 1:**
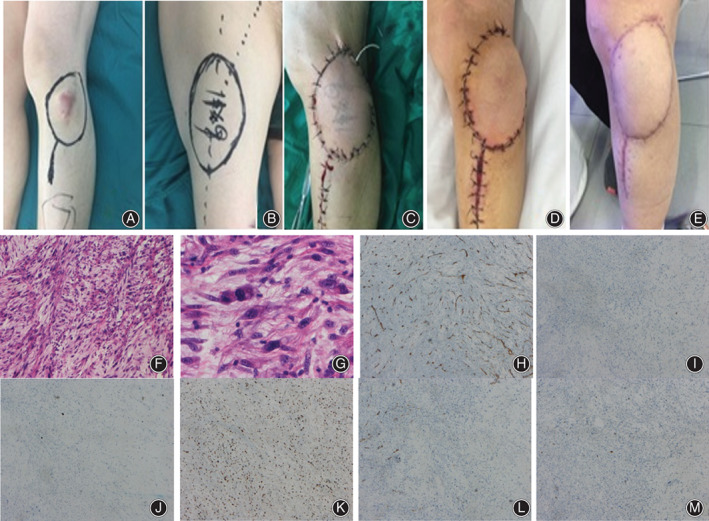
A patient undergoing reconstruction with free vascularized anterolateral thigh flap after wide resection of recurrent fibrosarcoma with follow‐up of 16 months (A). A Female patient, aged 67 years, was histologically diagnosed as recurrent undifferentiated high‐grade pleomorphic sarcoma located in the anterolateral knee. (A) The location of the recurrent fibrosarcoma with the first surgical scar and the planned wide excision territory. (B) The donor site of the free vascularized anterolateral thigh flap. (C) Postoperative picture after FVALTP was transferred and vascular anastomosis. (D) Postoperative picture two weeks later. (E) At the 16‐months follow‐up, the whole reconstruction was maintained well with no complication reported. The patient had excellent functional outcome with a MSTS score of 30. (F, G) H & E staining indicates spindle‐shaped cells and less often polygonal or epithelioid cells with more abundant cytoplasm. The pathological diagnosis is undifferentiated high‐grade pleomorphic sarcoma. (H–L). Immunohistochemical staining includes CD 34 (H), Desmin (I), EMA (J), Ki67 (K), S100 (L), and SMA (M). All these indexes appear negative except that Ki 67‐positive cell accounts for about 30%.

**FIG 2:**
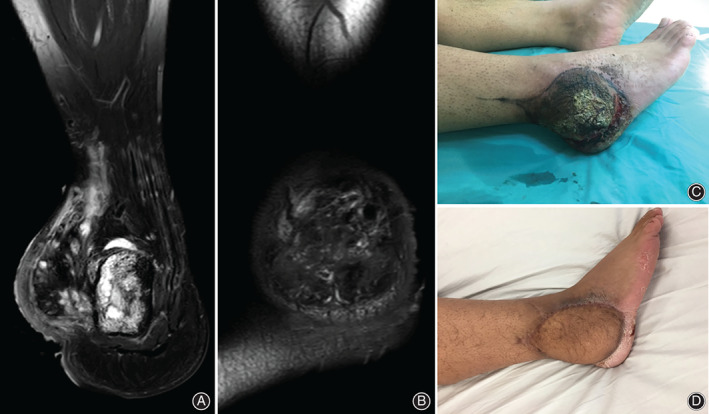
A patient undergoing reconstruction with free vascularized anterolateral thigh flap after wide resection of malignant peripheral nerve sheath sarcoma. (A) A male patient, aged 29 years, was histologically diagnosed as malignant peripheral nerve sheath sarcoma at left foot; (B) MRI showed the tumor; (C) preoperative picture of the tumor; (D) postoperative picture after FVALTP was transferred and vascular anastomosis.

**FIG 3:**
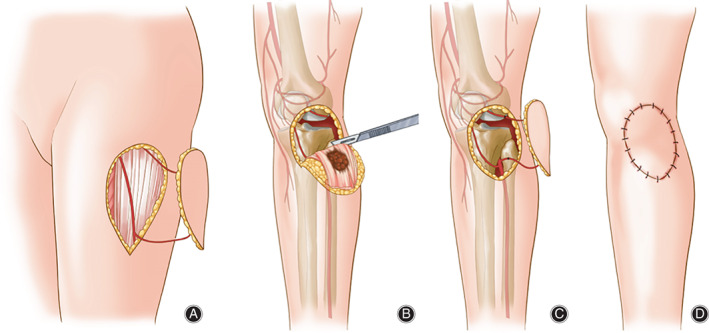
Illustration of surgical procedure: (A) harvest of FVALTP, (B) resection of soft tissue sarcoma, (C) The artery and vein were anastomosized with the local vessels near the recipient sites, (D) The flap is inset with a layered closure.

The standard surgical procedure is a wide excision with negative margins (R0, no residual microscopic disease) (Fig. 1) of at least 2 cm^10^. At half the distance between the anterior superior iliac spine and the superolateral patella, perforators are marked with the use of a handheld doppler. Perforators distal to this point are also interrogated to optimize pedicle length and/or flap size. A curvilinear medial incision is designed over the rectus femoris muscle, and dissection ensues medial to lateral in a subfascial plane until the corresponding marked perforators are identified. The rectus femoris muscle is then dissected circumferentially while preserving its major neurovascular pedicles and is then retracted medially. Depending on its course, the major flap perforator is freed from overlying fascia and/or muscle. Motor nerves are preserved if technically possible. Once the perforator(s) and the descending branch of the lateral circumflex femoral system are dissected circumferentially to the origin of the lateral circumflex system, the lateral incision of the skin paddle is made and the flap is islanded. The vascularized flap is then transferred to the recipient sites (Fig. 2). The artery and vein were then anastomosized with the local vessels near the recipient sites. The flap is inset with a layered closure (Fig. 3).

### Postoperative Management

Reconstructed limbs were immobilized with a cast for 3 weeks.

### Follow‐Up Analysis

Surgically related complications were recorded for each patient. During the follow‐up, computed tomography (CT) was routinely performed every 3 months. Functional outcomes of the reconstructed limbs were assessed with the MSTS scoring system.

### MSTS Scoring System

The MSTS score measures six domains including pain, function, emotional acceptance, supports, walking ability, and gait. A 5‐point scale was used for each domain, producing a maximum score of 30 points

### Statistical Analysis

Descriptive statistics, including the mean and standard deviation (SD) for continuous variables, and the frequency and proportion of categorical variables were calculated. Statistical analysis was performed using SPSS for Windows (version 22; SPSS Inc., Chicago, Illinois, US).

## Results

### Summary of Demographic Data

A total of 11 patients (four males and seven females) were included in the study. The mean age was 62 years (range: 29–84 years). The etiology was as follows: five cases of undifferentiated pleomorphic sarcoma, two cases of liposarcoma, one case of synovial sarcoma, one case of melanoma, one case of recurrent fibrosarcoma, and one case of peripheral malignant nerve sheath sarcoma. The mean surface area was 151.4 cm^2^ (range: from 64 cm^2^ to 418 cm^2^). The mean operation time was 126 min (range: 95–296 min).

### Clinical Outcomes

Incision healed by first intention in eight patients. Incision healed by second intention in three patients. The mean follow‐up was 17.5 months (range: 6–34 months). The mean score of MSTS at last follow‐up was 26.2 (range: 12–29).

### Complications

Four complications occurred in our cohort. A patient who had received preoperative radiotherapy experienced delayed union. After debridement, the patient successfully got union. Other two patients experienced marginal necrosis of flap due to vascular crisis. After 3‐week dressing changes, the patients also got satisfactory union. One case suffered from vascular crisis during surgery in which the procedure was changed into skin grafting to cover the resection site.

## Discussion

### Overview of Soft Tissue Reconstruction After Resection for STSs

In the past, sarcomas of the extremities were always treated by amputation^11^. The advent of multimodality treatment meant the amount of amputations was kept to a low level and the use of limb‐salvage techniques were popularized^12^. Rosenberg *et al*. compared limb‐sparing surgery plus RT with amputation and did not find any differences in overall survival rates or disease‐free survival rates^13^. The standard surgical procedure is a wide excision with negative margins (R0, no residual microscopic disease). To guarantee an R0 resection, the cutting face should go through grossly normal tissue planes uncontaminated by tumor^14^. One of the disadvantages of wide resection was soft tissue defect after surgery. Several local, regional, and distant flaps have to be used for coverage of defects. Pedicle skin flaps or local transposition flaps were traditionally used to repair soft tissue defects, but availability of local donor tissues is quite limited, especially when the defect was too large. In addition, when bone or tendon is exposed with gross tissue loss, local flaps cannot provide adequate coverage. Other goals for reconstructive surgery for defects after wide resection for sarcomas of extremities included: (i) a tendon reconstruction that could withstand the mechanical and shear forces required for normal function, such as standing, walking, and running; (ii) vascularized soft tissue that could fill a cavity, cover a wound, and protect healing and smooth movement of tendons; and (iii) durable skin coverage for an injured foot to wear shoes and move^15^. FVALTP perfectly meets the aforementioned requirements, and could preserve functions and restore cosmetic contour. It can be harvested with multiple tissue components including skin, fat, muscle, and fascia and is designed with a significant fascial component in the tensor fasciae latae, which is advantageous considering the recipient site in question. Moreover, some studies demonstrated more flexibility in using a composite of tissues including skin, muscle, and extensive fascia^16^.

### Complications of FVALTP

Four complications occurred in our cohort. A patient who had received preoperative radiation therapy experienced delayed union. Preoperative radiation was widely recognized as a risk factor of wound complication. It is recommended that an interval of 3–6 weeks is needed to decrease the risk of wound complications and to cool down the acute reactions. For this patient, we had left a 4‐week interval. However, wound complication still occurred. After debridement, the patient successfully got union. The other two patients experienced marginal necrosis of flap due to less blood supply. After 3‐week dressing changes, the patient also got satisfactory union. Vascular crisis was the major cause of necrosis of flap, and we prescribed papaverine to avoid vascular spasm, thus dramatically decreasing the incidence of vascular crisis^17^. Another case suffered from vascular crisis during surgery in which the procedure was changed into skin grafting to cover the resection site. Overall complication rate was lower in our cohort than in other literature. For other conditions, especially trauma‐related soft tissue defect, the preexisting necrosis and infection would make the rate of wound complication high^18^, ^19^.

Another question was whether age influences union of flap. In our cohort, age ranged from 29 to 84 years. We did not find much influence of age on surgical outcomes. The oldest patient in our cohort reached 84 years, and she got satisfactory union. Four wound complications were also not related to age, one for preoperative radiation therapy and the other two for vascular crisis.

### Limitation

The primary limitation lies in that this is small retrospective case series, as the incidence of STSs was quite low. It is noteworthy that all grafting procedures were carried out by a single surgeon (H. M.) who was experienced in microvascular techniques. All the reconstruction procedures were performed with the same protocol. Therefore, variations of surgical technique were minimized. Second, no control group was included in this study. In future study, other reconstruction techniques, such as local transfer flap, can be applied to better clarify the effectiveness of FVALTP.

In conclusion, FVALTP can successfully manage soft tissue defects after resection of sarcoma of extremities with a relatively low complication rate.
